# Hereditary Breast and Ovarian Cancer in Families from Southern Italy (Sicily)—Prevalence and Geographic Distribution of Pathogenic Variants in *BRCA1/2* Genes

**DOI:** 10.3390/cancers12051158

**Published:** 2020-05-05

**Authors:** Lorena Incorvaia, Daniele Fanale, Giuseppe Badalamenti, Marco Bono, Valentina Calò, Daniela Cancelliere, Marta Castiglia, Alessia Fiorino, Alessia Pivetti, Nadia Barraco, Sofia Cutaia, Antonio Russo, Viviana Bazan

**Affiliations:** 1 Department of Biomedicine, Neuroscience and Advanced Diagnostics (Bi.N.D.), Section of Medical Oncology, University of Palermo, 90127 Palermo, Italy; lorena.incorvaia@unipa.it; 2 Department of Surgical, Oncological and Oral Sciences, Section of Medical Oncology, University of Palermo, 90127 Palermo, Italy; fandan@libero.it (D.F.); giuseppe.badalamenti@unipa.it (G.B.); marcobono29@gmail.com (M.B.); valentinacalo74@gmail.com (V.C.); daniela.cancelliere83@gmail.com (D.C.); martacastiglia@gmail.com (M.C.); alessiafiorino94@gmail.com (A.F.); alessia_pivetti@libero.it (A.P.); barraconadia@gmail.com (N.B.); sofia.cutaia@gmail.com (S.C.); viviana.bazan@usa.net (V.B.)

**Keywords:** *BRCA1*, *BRCA2*, breast cancer, founder variants, genetic testing, germline pathogenic variants, hereditary breast and ovarian cancer, ovarian cancer, Sicilian population

## Abstract

Recent advances in the detection of germline pathogenic variants (PVs) in *BRCA1/2* genes have allowed a deeper understanding of the *BRCA*-related cancer risk. Several studies showed a significant heterogeneity in the prevalence of PVs across different populations. Because little is known about this in the Sicilian population, our study was aimed at investigating the prevalence and geographic distribution of inherited *BRCA1/2* PVs in families from this specific geographical area of Southern Italy. We retrospectively collected and analyzed all clinical information of 1346 hereditary breast and/or ovarian cancer patients genetically tested for germline *BRCA1/2* PVs at University Hospital Policlinico “P. Giaccone” of Palermo from January 1999 to October 2019. Thirty PVs were more frequently observed in the Sicilian population but only some of these showed a specific territorial prevalence, unlike other Italian and European regions. This difference could be attributed to the genetic heterogeneity of the Sicilian people and its historical background. Therefore hereditary breast and ovarian cancers could be predominantly due to *BRCA1/2* PVs different from those usually detected in other geographical areas of Italy and Europe. Our investigation led us to hypothesize that a higher prevalence of some germline *BRCA* PVs in Sicily could be a population-specific genetic feature of *BRCA*-positive carriers.

## 1. Introduction

Inherited pathogenic variants (PVs) in *BRCA1* or *BRCA2* genes cause a significantly increased risk for breast cancer (BC) [1], ovarian cancer (OC) [2] and other tumors including pancreas [3,4] and prostate cancer [5,6,7].

Although BC and OC are more frequently sporadic tumors (75–80%), approximately 15–20% is familial type and about 5–10% of cases is hereditary. *BRCA1* and *BRCA2* are the most known genes associated with hereditary breast and/or ovarian cancer (HBOC) risk [8]. However, the hereditary forms could be associated with germline PVs in several genes which confer a different risk to develop a neoplasm. PVs in genes such as *ATM, CHEK2, BARD1, BRIP1, RAD51C, RAD51D*, confer a moderate risk and, for this reason, the clinical management of PV carriers is, to date, still debated and requires further in-depth clinical studies [9,10,11]. PVs in *BRCA1/2, CDH1, PALB2, PTEN, STK11* and *TP53* genes confer a high risk to develop BC [12] but also OC or other tumors [13,14,15,16,17]. Specifically, *BRCA1* PV carriers show a 50–85% risk of developing BC during their lifetime, 40–60% risk of developing bilateral BC and 15–45% risk of develop OC or tubal carcinoma. Germline *BRCA2* PV carriers, like *BRCA1* PV carriers, exhibit a 50–85% risk of BC but a lower risk (10–20%) of developing OC [18,19].

*BRCA1*-associate BCs show frequently a poorly differentiated infiltrating ductal histotype, high proliferative activity (ki-67), negativity of hormone receptors for estrogens (ERs) and progesterone (PR) and absence of HER2/neu amplification (“triple-negative” phenotype, TNBC) [20]. These tumors are associated with higher mortality and morbidity and therefore involve only the use of conventional chemotherapy as therapeutic option. Instead, *BRCA2*-associated BCs have a molecular profiling that resembles the luminal A or B tumor subtypes [21,22] as well as a slight increase in the incidence of the lobular histotype compared to sporadic forms [23,24].

In the OC, the prevalence of *BRCA1/2* PVs is high (17–20%), especially in patients with serous tumors [25] and increases up to 23–25% in the high-grade serous histotype [26], independently of age of diagnosis and family history. For this reason, in the OC patients, according to the current guidelines, the *BRCA* testing is performed in all non-mucinous and non-borderline ovarian epithelial carcinoma, fallopian tube carcinoma and primary peritoneal carcinoma. The individuals and family members eventually *BRCA* PV carriers will be directed towards dedicated high-risk follow-up programs and specific risk-reducing strategies. Furthermore, today, genetic testing result has substantial clinical implications for patient care. In addition to preventive pathways for early diagnosis and cancer risk reduction developed for individuals at high-risk and their relatives, *BRCA* testing has become a predictive biomarker of therapy response [27].

Recently, several studies have demonstrated that the prevalence of germline PVs and gene-specific risk estimates could change, not only based on family history and type/molecular subtype of the tumors but also on the basis of race, ethnicity and different geographic location [28,29,30].

Although today population-based testing for hereditary BC and OC risk is not yet widespread, some studies evaluating population-based PVs mostly in the Jewish population are beginning to provide new information and new knowledge and, in the future, could lead to new directions in genetic testing in select groups of subjects [29,31,32]. Currently, the most used population model for studying the feasibility and efficacy of the population-based *BRCA* testing is represented by Jewish population; however, implementation studies are needed for testing multiple cancer susceptibility genes in the general population [29]. Therefore, the current guidelines for genetic testing, based on clinical criteria and family history, could be replaced by new approaches able to detect the 50% more *BRCA* carriers than those identified by conventional criteria [33,34].

In this context, little is known about the prevalence and geographic distribution of inherited PVs in *BRCA1/2* genes in the Sicilian population. For this purpose, in this work we performed a genetic screening for germline *BRCA1* and *BRCA2* PVs in Sicilian breast and/or ovarian cancer patients and their relatives, in order to assess the prevalence and typology of these high-risk susceptibility variants in individuals belonging to this specific geographical area of Southern Italy.

## 2. Results

### 2.1. Clinical Features of Breast and/or Ovarian Cancer Patients Harbouring Germline BRCA1/2 Pathogenic Variants

One thousand three hundred and forty six breast and/or ovarian cancer patients who met the previously established inclusion criteria (see Patients and Methods section) were recruited and studied over a period ranging from January 1999 to October 2019 at the “Regional Center for the prevention, diagnosis and treatment of rare and heredo-familial tumors of adults” of the Section of Medical Oncology of the University Hospital Policlinico “P. Giaccone” of Palermo. A substantial increase in the request and indication of *BRCA* testing for cancer risk assessment, resulting in gradual increase also in the number of PV carriers, was observed at our Centre during these years (Figure S1). In particular, a sharp increase in *BRCA* testing number was observed from 2016 to 2017, when *BRCA* testing has acquired a predictive value of response to the therapy by PARP inhibitors. This Guidelines change has allowed to extend the *BRCA* testing for therapeutic purposes to all patients with non-mucinous and non-borderline ovarian epithelial carcinoma, fallopian tube carcinoma and primary peritoneal carcinoma, also in absence of cancer family history.

**Figure 1:**
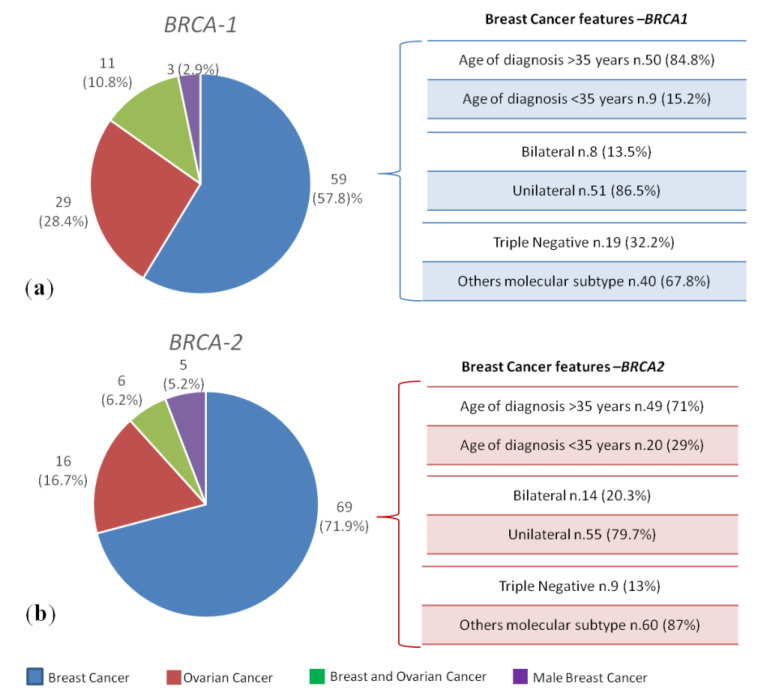
Breast and/or ovarian cancer patients harboring germline *BRCA1/2* pathogenic variants (PVs). 200 (14.8%) out of 1346 probands with breast and/or ovarian cancer had germline PVs in *BRCA1/2* genes. (**a**) 102 (51%) were *BRCA1* PV carriers, (**b**) 96 (48%) were *BRCA2* PV carriers and 2 patients (1%) showed a double heterozygosity for *BRCA1* and *BRCA2* PVs.

All 1346 probands, after appropriate genetic counselling, were genetically tested for germline PVs in *BRCA1* and *BRCA2* genes. 200 (14.8%) out of 1346 probands with breast and/or ovarian cancer have been shown to harbour germline *BRCA1/2* PVs. Our investigation showed that 102 (51%) out of 200 *BRCA* variant-positive individuals were *BRCA1* PV carriers, 96 subjects (48%) were *BRCA2* PV carriers and 2 patients (1%) showed a double heterozygosity for *BRCA1* and *BRCA2* PVs. Among 102 *BRCA1* PV carriers, 59 individuals (57.8%) had female breast cancer, 8 of which bilateral (13.5%), 19 triple-negative breast cancer (TNBC) (32.2%) and 9 diagnosed before age 35 years (15.2%), 29 patients (28.4%) had ovarian cancer, 27 of which high-grade serous (93.1%) and 2 endometrioid (6.9%), 11 subjects (10.8%) had both primary ovarian and breast cancer, 3 patients (2.9%) had male breast cancer (Figure 1a; Table 1). 

Among 96 *BRCA2* PV carriers, 69 individuals (71.9%) had female breast cancer, 14 of which bilateral (20.3%), 9 TNBC (13%) and 20 diagnosed before age 35 years (29%), 16 patients (16.7%) had ovarian cancer, 15 of which high-grade serous (93.7%) and 1 endometrioid (6.3%), 6 subjects (6.2%) had both primary ovarian and breast cancer, 5 patients (5.2%) had male breast cancer (Figure 1b; Table 1). 

Finally, two patients showing a double heterozygosity for *BRCA1* and *BRCA2* PVs were probands with BC, one of which TNBC and the other bilateral (luminal B subtype). 

In our study, TNBCs account for 25% of all *BRCA*-variant breast cancers present in probands: 70% of these was related to *BRCA1* PVs and 30% was associated to *BRCA2* PVs. Also, it has been observed that 37% of genetically tested TNBC patients was carrier of germline PVs in *BRCA* genes.

### 2.2. Clinical Features of Germline BRCA1/2 PV Carriers

Afterwards, in our study we have also included the relatives of *BRCA*-positive probands enrolled at our Centre in the same period of time ranging from January 1999 to October 2019. Six hundred and twenty one family members were enrolled and tested for mutational analysis in *BRCA1/2* genes and 285 (45.9%) out of the 621 family members have been shown to be carriers of a *BRCA1/2* PV. Considering both probands and family members, overall 1967 individuals were genetically tested for *BRCA1/2* PVs at our Centre. Our analysis showed that 485 (24.6%) out of 1967 enrolled subjects (probands and family members) harbor a germline *BRCA1/2* PV (Figure S2).

**Figure 2:**
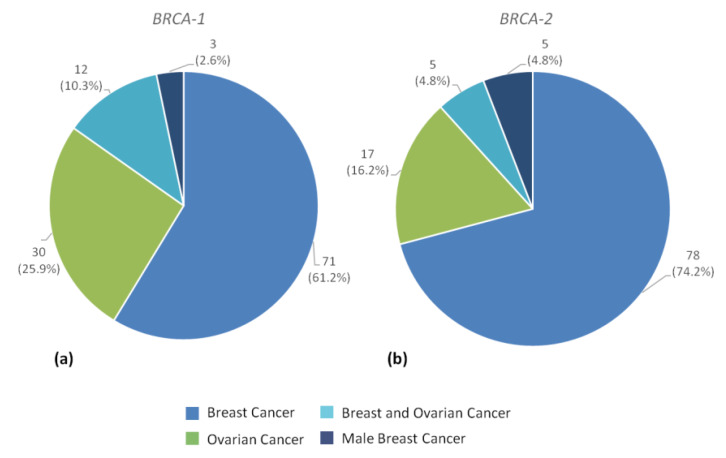
Type of tumors in all individuals carrier of germline *BRCA1* (**a**) or *BRCA2* (**b**) PVs (probands and family members).

Out of 485 individuals, 259 (53.4%) were *BRCA1* PV carriers, 222 subjects (45.8%) were *BRCA2* PV carriers and 4 individuals (0.8%) showed a double heterozygosity for *BRCA1* and *BRCA2* PVs. Among 259 *BRCA1* PV carriers, 116 individuals (44.8%) were patients with cancer diagnosis related to hereditary breast and/or ovarian cancer syndrome, 133 subjects (51.3%) were healthy and 10 individuals (3.9%) were excluded from the study for lack of sufficient information. Of 116 cancer patients, 71 (61.2%) had female breast cancer, 11 of which bilateral (15.5%) and 8 diagnosed before age 35 years (11.3%), 30 patients (25.9%) had ovarian cancer, 12 patients (10.3%) had both primary ovarian and breast cancer and 3 patients (2.6%) had male breast cancer (Figure 2a, Table 2). 

Among 222 *BRCA2* PV carriers, 105 individuals (47.3%) were cancer patients, 109 subjects (49.1%) were healthy and 8 individuals (3.6%) were excluded from the study for lack of sufficient information. 78 (74.2%) out of 105 cancer patients had female breast cancer, 15 of which bilateral (19.2%) and 20 diagnosed below age 35 years (25.6%), 17 patients (16.2%) had ovarian cancer, 5 patients (4.8%) had both primary ovarian and breast cancer and 5 patients (4.8%) had male breast cancer (Figure 2b, Table 2). 

Finally, 2 out of 4 subjects showing a double heterozygosity for *BRCA1* and *BRCA2* PVs were probands with BC and the other two were healthy family members.

### 2.3. Typology and Distribution of BRCA1/2 PVs in the Sicilian Population

Our investigation mainly aimed to assess the typology and prevalence of germline *BRCA1* and *BRCA2* PVs in individuals belonging to different geographical areas of Sicilia. Mutational analysis revealed that 99 *BRCA1/2* variants (classes III and V), 58 of which in *BRCA2* and 41 in *BRCA1*, were present in the 1346 genetically tested probands. No large rearrangements were detected in the 1346 probands through CNV analysis. Based on the classification criteria developed by the Evidence-based Network for the Interpretation of Germline Mutant Alleles (ENIGMA) consortium (https://enigmaconsortium.org/) and according to IARC recommendations [35], 64 out of 99 *BRCA1/2* variants were classified as pathogenic (class V) and 35 were variants of uncertain significance (VUS; class III) (Tables S1 and S2). Among the 202 PVs identified in the patient cohort, that also included 4 PVs harbored by two probands with double heterozygosity for *BRCA1* and *BRCA2*, 64 were different PVs. Thirty most recurrent germline PVs were observed in the Sicilian population. The most frequent germline *BRCA* PVs present in the Sicilian population are reported in Table 3 and Table 4. 

The most frequent PV identified in most *BRCA*-positive carriers is named *BRCA1*-5083del19 (HGVS nomenclature: c.4964_4982del; p.Ser1655fs). The analysis of the haplotype of carriers of this PV showed that these subjects originate from a common ancestor [36,37,38,39]. This variant was observed in several families with breast and ovarian cancer and was first described as a Calabrian/Southern Italian founder variant [40] but today it is considered a potential Sicilian founder mutation [41]. Thirteen percent of all *BRCA*-positive carriers was carrier of this germline PV, which was detected in 18 Sicilian families, involving a total of 63 carriers (18 probands, 45 family members), of which 25 cancer patients (39.68%). Among cancer patients harboring this founder variant, 12 individuals had breast cancer, 12 ovarian cancer and one patient only had both primary ovarian and breast cancer. This founder variant was mainly observed in several families from the Western Sicily, especially in the geographical areas including the cities of Palermo (located on the northern coast of Sicily) and Agrigento (located on the southern coast of Sicily) and respective neighboring areas. 

Another less recurrent Sicilian PV with potential “founder effect” is named *BRCA1*-4843delC (HGVS nomenclature: c.4583del; p.Pro1528fs) [42] and was detected in 7 individuals only (2 of which were BC patients) from the city of Palermo. 

The second most frequent PV, found in 12 families and involving a total of 49 subjects (10.1%), including 20 cancer patients (12 probands and 8 family members), is named *BRCA2*-1466delT (HGVS nomenclature: c.1238del; p.Leu413fs) [43,44,45,46]. Most of the Sicilian cancer patients harboring this PV had breast cancer (19 subjects) and one only ovarian cancer. This sequence variant was specifically observed in several families from North-Western Sicily, particularly in the geographical area including the city of Palermo and neighboring areas (for example, the small town of Montemaggiore Belsito). 

The third most frequent PV, observed in 14 families and involving a total of 38 subjects (8%), including 15 cancer patients (14 probands and 1 family member), is named *BRCA1*-633delC (HGVS nomenclature: c.514del; p.Gln172fs) [39,47]. Among Sicilian cancer patients harboring this PV, 8 individuals had breast cancer, 6 ovarian cancer and one patient only had both primary ovarian and breast cancer. This PV was mainly observed in some subjects from the Northern Sicily, especially in the geographical areas including the cities of Palermo (western coast of Sicily) and Messina (eastern coast of Sicily) and respective neighboring areas.

A PV, mainly observed in some families from North-Western Sicily (16 individuals, 5 of which cancer patients) belonging to the geographical area that includes the city of Trapani and neighboring areas, is named *BRCA2*-6310del5 (HGVS nomenclature: c.6082_6086del; p.Glu2028fs) [48,49]. Among cancer patients harboring this PV, 3 subjects had breast cancer, 1 ovarian cancer and 1 both primary ovarian and breast cancer. 

Furthermore, another PV named *BRCA2*-6079del4 (HGVS nomenclature: c.5851_5854del; p.Ser1951fs) [50,51,52] was also detected in some families from North-western Sicily (17 individuals, including 8 cancer patients) belonging to the geographical area that includes the cities of Palermo and Trapani and neighboring areas. Among cancer patients harboring this PV, 5 individuals had female breast cancer, 2 male breast cancer and one both primary ovarian and breast cancer. Another PV named *BRCA2*-K2013X (HGVS nomenclature: c.6037A > T; p.Lys2013Ter) was exclusively detected in two Sicilian families (five PV carriers) from the city of Erice (North-western Sicily). 

Other PVs observed with lower recurrence in Sicilian families belonging to the geographical area that includes the city of Palermo and neighboring areas are the following: *BRCA1*-1479delAG (HGVS nomenclature: c.1356_1357AG [2]; p.Glu453_Ser454insTer), *BRCA1*-R1443X (HGVS nomenclature: c.4327C > T; p.Arg1443Ter), *BRCA1*-5149del4 (HGVS nomenclature: c.5030_5033del; p.Thr1677fs), *BRCA1*-3347delAG (HGVS nomenclature: c.3226_3227AG [1]; p.Gly1077fs), *BRCA1*-916delTT (HGVS nomenclature: c.798_799del; p.Ser267fs). Interestingly, *BRCA1*-R1443X has been reported as a founder variant in the French Canadian population [53], *BRCA1*-3347delAG as a founder mutation both in Italy (Tuscany) and Norway [38], *BRCA1*-916delTT as a North African founder variant [54].

The three most represented PVs, observed in families from the Sicilian hinterland, especially in those belonging to the geographical area that includes the city of Caltanissetta and neighboring areas (for example, the small town of Mussomeli), are named *BRCA1*-5149del4 (HGVS nomenclature: c.5030_5033del; p.Thr1677fs), *BRCA1*-E1302X (HGVS nomenclature: c.3904G > T; p.Glu1302Ter), *BRCA2*-3036del4 (HGVS nomenclature: c.2808_2811del; p.Ala938Profs). Interestingly, *BRCA2*-3036del4 has been described as a founder variant in Western Europeans [55].

Furthermore, a common Ashkenazi founder PV consisting of a *BRCA1* duplication named 5382insC (HGVS nomenclature: c.5266dupC; p.Gln1756Profs) [56] has been mainly observed in some families from the North-eastern coast of Sicily belonging to the city of Messina (11 individuals, including 6 cancer patients). Among cancer patients harboring this PV, 5 subjects had breast cancer (3 of which TNBC) and 1 had ovarian cancer. Interestingly, this variant was reported as a founder mutation also in several European populations (Austrian, Slovenian, German, Czech, Hungarian, Greek, Danish, Polish, Latvian, Lithuanian, Belarusian and Russian) [53].

In addition, another common Ashkenazi founder PV named *BRCA1*-185delAG was detected in one Sicilian family (3 individuals, one of which with BC) from the city of Ragusa (Table S1).

Another recurrent founder PV, named *BRCA1*-C61G (HGVS nomenclature: c.181T > G; p.Cys61Gly) and identified mainly in Eastern European populations (Polish, Czech, Slovenian, Austrian, Hungarian, Belarusian and German) [38], has also been observed in our study population (8 PV carriers, including 2 cancer patients). Also the *BRCA2*-9326insA founder variant, usually present in Germany and Hungary [38], was detected in Sicilian individuals (7 PV carriers, 2 of which cancer patients) from the cities of Palermo and Agrigento. Conversely, the *BRCA2*-9254del5 founder mutation originating in the Northeast Spanish [57] was observed in 6 Sicilian subjects, including 3 cancer patients, from the city of Palermo. Finally, two PVs named *BRCA1*-3372insA (HGVS nomenclature: c.3253dupA; p.Arg1085Lysfs) and *BRCA2*-IVS21 + 4A > G (HGVS nomenclature: c.8754 + 4A > G) have been detected in some families belonging to the geographical area that includes the city of Catania and neighboring areas.

**Figure 3:**
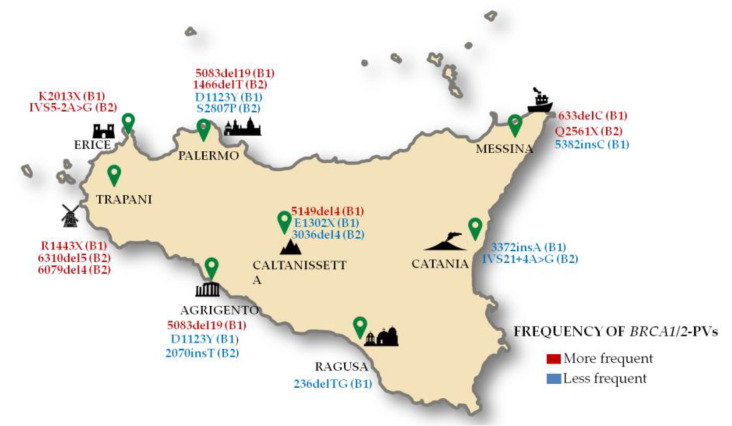
Geographical distribution of the hereditary breast/ovarian cancer families in Sicily.

The distribution of the most frequent *BRCA1/2* PVs in different geographical areas of Sicily is shown in Figure 3.

## 3. Discussion 

In the last years, thanks to advances in genetic sequencing techniques, the field of genetic testing for breast and ovarian cancers has deeply evolved. Our improved ability of profiling the molecular and genetic features of tumors has allowed for a better and more accurate detection of germline but also somatic, PVs in several genes, including *BRCA1/2*, resulting in a deeper understanding of the cancer risk related to heredo-familial tumor syndromes. Germline *BRCA* PVs occur in 10–20% of TNBC patients and are especially common among BC patients with *BRCA1* PV than *BRCA2* PV carriers or non-carriers. 

In our work, we have retrospectively collected and analyzed all clinical information of 1346 HBOC patients enrolled from January 1999 to October 2019 at our Centre and genetically tested for germline PVs in *BRCA1* and *BRCA2* genes. Our study has mainly focused on both the epidemiological impact of the germline *BRCA* PVs found in the Sicilian population and geographical distribution of these variants. The comparison between all genetically tested individuals (probands and family members) harboring PVs in *BRCA1* and *BRCA2* genes showed that there is no a clear prevalence of variants in one gene rather than the other in the Sicilian population, confirming the epidemiological and genetic data previously reported in literature. Specifically, 53% of analyzed *BRCA*-positive subjects carried a *BRCA1* PV, 46% harbored a *BRCA2* PV and 1% was characterized by simultaneous presence of PVs in *BRCA1* and *BRCA2* genes. As regards the typology of cancer associated with HBOC syndrome in the *BRCA1* PV carriers, 60% of tumors was represented by female BC, 26% by OC, 10% by both (primary BC and OC) and finally 3% by male BC. Instead, *BRCA2* PV carriers showed female BC in 74% of cases, OC in 16%, primary BC and OC in 5% and male BC in remaining 5%. By comparing the previous data, we have observed that *BRCA1* PV carrier patients show a higher frequency of OC (26% vs. 16%) and synchronous or metachronous primary BC and OC, compared to *BRCA2* PV carrier patients. Conversely, *BRCA2* PV carrier patients have been observed to exhibit a higher incidence of female BC (74% vs. 60%) characterized by earlier age of onset (diagnosis under age 35 years: 26% vs. 11%), bilateral female BC and male BC, compared to *BRCA1* PV carrier patients. Furthermore, it has been observed that the mean and median ages of onset for BC were similar for PV carriers in both genes. Instead, *BRCA2* PV carrier patients showed, not only a lower frequency of OC but also a later onset age of disease (mean age at diagnosis: 60.6y vs. 52y; median age at diagnosis: 57y vs. 51y), compared to the *BRCA1* PV carrier patients.

Subsequently, we investigated the prevalence and geographic distribution of inherited PVs in *BRCA1/2* genes in the Sicilian population.

Several studies have reported numerous differences and a significant heterogeneity in the prevalence of PVs across different populations [34,58]. Based on these data, the cancer risk estimates could also be modified by race and ethnic origin. “Founder” mutations, originated from an ancestor population and maintained over time, were observed in specific geographic areas [59]. Significant evidence from founder mutation testing carried out mainly on the Jewish population allowed to develop new population-based genetic approaches that, in the future, could help to increase the *BRCA* carrier detection rates and maximize prevention strategies [34].

DNA sequence variants with “founder effect” were observed, for the first time, in Ashkenazi Jews, who have been shown to harbor a *BRCA1* PV named 185delAG in 1% of cases, resulting in a 16–20% BC risk below the age of 50 years [60]. Afterwards, other founder mutations have also been identified in various European populations [38,61]. Two PVs named *BRCA1*-5083del19 and *BRCA2*-8765delAG have shown a significant prevalence in Italian population [62,63]. The *BRCA1*-5083del19 PV was identified as founder mutation in families of Calabrian origin and, more recently, in numerous families of Sicilian origin [41]. Instead, *BRCA2*-8765delAG, detected in families with multiple cases of HBOC, was reported as a founder PV for several populations, including Northern Sardinian, French Canadians, Jewish-Yemenites and Czechs [62]. Several evidence supports the hypothesis that the 8765delAG variant occurred at least twice in different populations, since its position in an AG-rich sequence may represent a mutational hot-spot [62]. Finally, the PV named *BRCA1*-1499insA is considered a hypothetical founder variant in Tuscany [64]. 

Ninety-nine germline *BRCA1/2* variants, 58 of which in *BRCA2* and 41 in *BRCA1*, have been identified in the Sicilian families genetically tested in our study by mutational screening. We have observed that 30 PVs were more frequently detected in the examined Sicilian population but only some of these appeared more interesting in terms of territorial prevalence. As already previously reported [41], the *BRCA1*-5083del19 founder variant is resulted to be the most widespread PV in the Sicilian population, since it has been detected in 18 Sicilian families for a total of 63 carriers. This variant appears to be mainly widespread in the Western Sicily, in particular in the geographical areas that include Palermo and nearby cities (Bagheria, Roccamena, etc.) and Agrigento and neighboring towns (Sambuca, Sciacca, Santa Margherita di Belice). However, a haplotype analysis is required in all *BRCA1*-positive carriers present in Italy, in order to explain whether the Calabrian and Sicilian families have a common ancestor.

Conversely, the other potential Sicilian founder variant named *BRCA1*-4843delC seems to be poorly represented in the Sicilian population, because it has been detected only in 7 individuals from the city of Palermo. 

Also other two PVs named *BRCA2*-1466delT and *BRCA1*-633delC are resulted to be prevalent in the Sicilian population, because they have been observed in 12 (49 carriers) and 14 (38 carriers) families, respectively. Interestingly, since these two PVs have been rarely observed in other Italian regions or in the world, therefore they could be worthy of a more in-depth study in order to establish whether or not they are variants specific for the Sicilian population.

Almost all patients who harbored the *BRCA2*-1466delT variant showed a higher genetic predisposition to developing BC. This is a PV mainly detected in several families from the North-Western Sicily, especially in the geographical area that includes the city of Palermo and nearby areas. Interestingly, this variant seems to be very frequent mostly in Sicilian families from the small town of Montemaggiore Belsito.

The *BRCA1*-633delC variant was mainly observed in some families from the Tyrrhenian side of Sicily, especially in the geographical areas that include the cities of Palermo (western coast) and Messina (eastern coast) and respective neighboring areas.

Furthermore, other PVs, such as *BRCA2*-6310del5, *BRCA1*-E1302X, *BRCA1*-3372insA, *BRCA2*-K2013X and *BRCA2*-2070insT, deserve a more in-depth study not so much for a significant numerical incidence as for a specific territorial prevalence and segregation. Indeed, also these PVs have not been frequently detected in other countries or regions. 

The *BRCA2*-6310del5 PV has been mainly detected in some families belonging to the geographical area that includes the city of Trapani (North-Western Sicily), whereas the *BRCA2*-K2013X variant has been exclusively observed in some individuals from the city of Erice, a small town near Trapani.

The *BRCA1*-E1302X PV is resulted to be prevalent in a few families from the Sicilian hinterland, especially in the geographical area to which Caltanissetta belongs together with the nearby small town of Mussomeli. *BRCA1*-3372insA and *BRCA2*-2070insT, instead, seem to be PVs exclusive of the geographical areas that include the city of Catania (particularly, the smalls towns of Fiumefreddo di Sicilia and Aci Sant’Antonio) and Agrigento (especially, the small town of Raffadali), respectively. Finally, the *BRCA2*-V211I PV did not show territorial prevalence, but, in all cases examined in this study, is resulted to be associated with the emergence of bilateral female BC. 

In conclusion, our study showed that, unlike other Italian and European regions, some PVs observed in the Sicilian population have been shown to be more recurrent. This difference could be attributed to several factors including, for example, the historical background of the Sicily and its crucial geographical location in the center of the Mediterranean Sea, crossroads of several cultures and civilizations. In fact, the different peoples, such as Greeks, Romans, Arabs, Normans, Phoenicians, Etruscans, Byzantines, who dominated this region throughout history are probably responsible for the genetic heterogeneity of the Sicilian families, already reported for allelic variants of hemoglobin in Beta Thalassemia [65]. A further proof of this genetic heterogeneity reflecting the coexistence of different past cultures in Sicily may be provided by the presence in some Sicilian families of some specific founder variants observed in other countries (mostly Europe). In this context, for example, interestingly, two common Ashkenazi founder PVs, named *BRCA1*-5382insC and *BRCA1*-185delAG, were also observed in our study population, albeit with different frequencies.

The development of new population-based genetic approaches may help to detect the 50% more *BRCA*-carriers than those identified by classical clinical and familial criteria, as already shown in other studies [34].

Additionally, a more in-depth understanding of the correlations between described *BRCA* PVs and clinical features of HBOC syndrome patients could be the key to develop tailored treatment for *BRCA*-related cancer patients and better strategies for prevention and surveillance of *BRCA*-positive carriers without disease. Finally, several studies showed the association between specific *BRCA1/2* PVs and variations of breast and ovarian cancer relative risks [66]. Further investigations on this issue could help to better understanding the pression of genotype on tumor phenotype, favoring the development of tailored strategies for prevention, surveillance and treatment.

## 4. Materials and Methods

### 4.1. Study Population

This is a retrospective study based on *BRCA1/2* genetic testing results and clinical record review. We reviewed genetic, clinical and demographic data of patients with breast and/or ovarian cancer who underwent *BRCA1/2* genetic testing from January 1999 to October 2019 at the “Sicilian Regional Center for the Prevention, Diagnosis and Treatment of Rare and Heredo-Familial Tumors” of the Section of Medical Oncology of University Hospital Policlinico “P. Giaccone” of Palermo. Genetic counselling was performed by a multidisciplinary team, consisting mainly of an oncologist, a geneticist and a psychologist, who has collected all information regarding the personal and familial history of patients. For each family, the youngest member with breast and/or ovarian cancer has been selected as index case for the mutational screening. The information about cancer diagnosis and histological subtype was retrieved by pathology reports. Following the genetic counselling for evaluation of risk of HBOC syndrome, the patients were selected for germline *BRCA1/2* testing based on probability rate of carrying PV assessed by BRCAPRO genetic risk prediction model [67] and according to the previously established criteria by the Italian Association of Medical Oncology (AIOM). (https://www.aiom.it/linee-guida-aiom-2018-neoplasie-della-mammella-11/). These criteria are based on personal and family history and age of cancer onset, in order to identify individuals at high risk of harboring a PV in the HBOC predisposition genes. The following criteria were used to select patients to be genetically tested for germline *BRCA1/2* PVs: (i) Personal history of (a) male with BC; (b) women with breast and ovarian cancer; (c) woman with BC <36 years; (d) woman with TNBC <60 years (starting from year 2017); (e) woman with bilateral BC <50 years; (ii) Personal history of BC <50 years and at least 1 first-degree relative with: (a) BC <50 years; (b) non-mucinous and non-borderline OC at any age; (c) bilateral BC; (d) male BC; (iii) Personal history of BC >50 years and family history of breast or ovarian cancer in two or more relatives who have a first degree relationship with each other (including 1 who has a first-degree relationship with her); (iv) Family history of known PV in a predisposing gene [27]. The Italian Association of Medical Oncology (AIOM) every year updates its guidelines for identifying the individuals who should receive *BRCA* genetic testing, and, in the last version published at October 2019, AIOM expanded the population to be genetically tested, including the patients with advanced pancreatic cancer (https://www.aiom.it/wp-content/uploads/2019/10/2019_Racc_BRCA_pancreas.pdf). 

All the most relevant genetic, clinical and demographic information, including age of cancer diagnosis, histological subtype, personal and familial history and family geographical origin, was anonymously recorded and coded for all tested patients who previously provided written informed consent. 

The *BRCA* test result was considered informative, when a pathogenic or likely pathogenic variant was identified in an individual. Conversely, *BRCA* test result was considered not informative, when no pathogenic or likely pathogenic variant was identified but its presence could not be excluded or a VUS to which it was not possible to attribute a risk value was detected. 

Patients harboring a germline PV in *BRCA1/2* genes were addressed to enhanced screening programs and/or risk-reducing surgical strategies by a professional with expertise in cancer genetics. Targeted *BRCA1/2* testing was proposed and extended to the first-degree family members of *BRCA*-variant patients, after providing informed consent. 

Geographical origin of the examined Sicilian families was assessed based on the place of birth of index cases and tested family members. Afterwards, individuals harboring a *BRCA1/2* PV were divided into different geographical groups based on their original place of birth, considering the main cities of Sicily (Palermo, Catania, Messina, Agrigento, Caltanissetta, Trapani, etc.) and respective neighboring areas. Subjects from neighboring small towns have been included in the same geographical area of respective main city.

### 4.2. Sample Collection and Next-Generation Sequencing Analysis for BRCA 1/2 Genes

Peripheral blood samples were collected from HBOC patients. Genomic DNA was isolated from the peripheral blood using the DNeasy^®^ Blood Kit (QIAGEN, Hilden, Germany), quantified by Qubit^®^3.0 fluorometer (Thermofisher Scientific, Waltham, MA, USA) and its quality was assessed by using 2100 Bioanalyzer (Agilent Technologies, Santa Clara, CA). We used 4 ng of DNA to prepare the barcoded library using *BRCA* Screen kit (4bases SA) that has allowed to investigate all the exons of *BRCA1* (NM_007300.3) and *BRCA2* (NM_000059.3) genes. The kit consists of three multiplex PCR primer pools. We used 20 nanograms of DNA per primer pool for multiplex PCR amplification, followed by ligation of a barcode and purification with Agentcourt AMPureXP reagent (Beckman Coulter, Beverly, MA, USA). The quantity and the quality of prepared libraries were evaluated using Qubit^®^3.0 fluorometer (Thermofisher Scientific) and Agilent 2100 Bioanalyzer on-chip electrophoresis (Agilent Technologies), respectively, as previously described [68]. Subsequently, libraries were mixed in an equimolar ratio and emulsion PCR was performed with the Ion OneTouch OT2 System (Thermofisher Scientific) using Ion 520 & Ion 530 Kit-OT2 (Thermofisher Scientific). Finally, sequencing was performed with Ion 520 Chip (Thermofisher Scientific) using Ion Torrent S5 (Thermofisher Scientific) instrument. The sequencing data was analyzed with Amplicon Suite (SmartSeq s.r.l.) and Ion Reporter Software v.5.12 (Thermofisher Scientific).

### 4.3. Sanger Sequencing

Pathogenic, likely pathogenic and VUS variants of *BRCA1/2* genes were confirmed by Sanger sequencing using a BigDye Therminator 3.1 Cycle Sequencing Kit (Life Technologies) and read through the 3130xl Genetic Analyzer (Applied Biosystems), according to the manufacturer’s protocols.

### 4.4. CNV Analysis by Multiplex Ligation-Dependent Probe Amplification Analysis (MLPA)

The presence of Large Genomic Rearrangements (LGR) was additionally tested by Multiplex ligation-dependent probe amplification (MLPA), using the SALSA MLPA probemix P002-C2 for *BRCA1* gene and SALSA MLPA Probemix P090 for *BRCA2* gene according to the manufacturer’s instructions (MRC–Holland, Amsterdam, the Netherlands). Probe amplification products were analyzed by capillary electrophoresis using ABI 3130 Genetic Analyzer (Applied Biosystems). Results were analyzed by GeneMapper™ Software Version 3.5 (Applied Biosystems) to determine peak heights and areas and fragment sizes in base pairs (bp), as described previously [69]. Positive results were confirmed with an additional analysis using the same kit on a second blood sample.

### 4.5. Genetic Variant Classification

The *BRCA* genetic variants were screened based on the classification criteria developed by the Evidence-based Network for the Interpretation of Germline Mutant Alleles (ENIGMA) consortium (https://enigmaconsortium.org/) and according to IARC recommendations [35], using a system of division into five classes—benign (class I), likely benign (class II), variant of uncertain significance (VUS, class III), likely pathogenic (class IV) and pathogenic (class V). Several databases, such as ClinVar, *BRCA* Exchange, LOVD, were used for the search and classification of *BRCA* variants.

The *BRCA* PVs identified in *BRCA*-positive carriers were named according to the systematic nomenclature of The Breast Cancer Information Core (BIC) database (http://research.nhgri.nih.gov/bic/) [70] and to the recommendations for the description of sequence variants established by the Human Genome Variation Society (HGVS). HGVS nomenclature was authorized by the HGVS, Human Variome Project (HVP) and the Human Genome Organization (HUGO) [71].

## 5. Conclusions

Since the Sicilian population is genetically different from the peoples of Europe and Northern Italy, probably due to its historical background, hereditary breast and ovarian cancers could be predominantly caused by *BRCA1/2* PVs less recurrent than those usually detected in other geographical areas of Italy and Europe. 

Our investigation has led us to hypothesize that a higher prevalence of some germline *BRCA* PVs in Sicilian families could be a population-specific genetic feature of *BRCA*-positive carriers.

## Figures and Tables

**Table 1:** Number of patients with Hereditary Breast/Ovarian Cancer (HBOC) and *BRCA1/2* pathogenic variants (PVs) (1999–2019), type of tumors (breast cancer, ovarian cancer, breast and ovarian cancers, male breast cancer), median age at diagnosis and family history.

*BRCA1/2* PVs Positive/Total HBOC *n* = 200/1346 (14.8%)	*BRCA1* PVs Positive *n* = 102 (51%)				*BRCA2* PVs Positive *n* = 96 (48%)				DH *BRCA1-* *BRCA2* PVs *n* = 2 (1%)
**Type of cancer**	Breast cancer *n* = 59 (57.8%)	Ovarian cancer *n* = 29 (28.4%)	Breast and ovarian cancers *n* = 11 (10.8%)	Male breast cancer *n* = 3 (2.9%)	Breast cancer *n* = 69 (71.9%)	Ovarian cancer *n* = 16 (16.7%)	Breast and ovarian cancers *n* = 6 (6.2%)	Male breast cancer *n* = 5 (5.2%)	Bilateral Breast cancer *n* = 2 (100%)
**Age at diagnosis (yr), median (range)**	41 (28–62)	52 (39–69)	BC 48 (36–77)	60 (67–73)	41 (25–80)	59 (40–81)	BC 50 (39–78)	67 (62–68)	Patient 1 37,41
			OC 53 (39–78)				OC 57 (41–79)		Patient 2 54,54
**Family history** **Yes, *n* = 53 (26.5%)** **No, *n* = 147 (73.5%)**	20 (33.8%) 39 (66.2%)	7 (24.1%) 22 (75.9%)	2 (18%) 9 (82%)	0 (0%) 3 (100%)	16 (23.2%) 53 (76.8%)	4 (25%) 12 (75%)	2 (33.3%) 4 (66.7%)	0 (0%) 5 (100%)	2 (100%) 0 (0%)

Abbreviations: BC = Breast Cancer; OC = Ovarian Cancer; PVs = Pathogenic Variants; HBOC= Hereditary Breast/Ovarian Cancer; DH = Double Heterozygosity.

**Table 2:** Total number of tested individuals (probands and family members), number of individuals carrier of germline *BRCA1/2* PVs and type of tumors.

*BRCA1/2* PVs Positive *n* = 485/1967 (24.6%).	*BRCA1* PVs Positive *n* = 259/485 (53.4%)				*BRCA2* PVs Positive *n* = 222/485 (45.8%)				DH *BRCA1-2 * PVs *n* = 4/485 (0.8%)
**No evidence of cancer *n*.244**	133 (51.3%)				109 (49.1%)				2 (50%)
**Lack of information *n*.18**	10 (3.9%)				8 (3.6%)				--
Diagnosis of cancer *n*.223	116 (44.8%)				105 (47.3%)				2 (50%)
Type of cancer	BC 71 (61.2%)	OC 30 (25.9%)	BC and OC 12(10.3%)	MBC 3 (2.6%)	BC 78 (74.2%)	OC 17 (16.2%)	BC and OC 5(4.8%)	MBC 5 (4.8%)	BC 2 (100%)

Abbreviations: BC = Breast Cancer; OC = Ovarian Cancer; MBC = Male Breast Cancer; PVs = Pathogenic Variants; DH = Double Heterozygosity.

**Table 3:** Type and frequency of germline *BRCA1* PVs (class V) and geographical area of origin of *BRCA* carriers.

BIC NOMENCLATURE	HGVS NOMENCLATURE	TYPE OF *BRCA1* PV	*BRCA1* PV CARRIERS (Patients and Family Members)	*BRCA1* PV Cancer Patients	ALLELE FREQUENCY (ExAC */GnomAD **)	ALLELE FREQUENCY (Sicilian Population)	GEOGRAPHICAL AREA
**5083del19 (S551fs)**	c.4964_4982del (p.Ser1655fs)	Deletion	63 (13.0%)	25 (39.68%)	ExAC 0.000008	0.00928	Palermo and Agrigento
**633delC (Q172fs)**	c.514del (p.Gln172fs)	Deletion	38 (7.83%)	15 (39.47%)	ExAC 0.000008 GnomAD 0.000004	0.00557	Palermo and Messina
**1479delAG**	c.1356_1357AG [2] (p.Glu453_Ser454insTer)	Deletion	14 (2.87%)	6 (42.86%)	ExAC 0.000016 GnomAD 0.000008	0.00222	Deletion
**4446C > T (R1443X)**	c.4327C > T (p.Arg1443Ter)	SNV	12 (2.48%)	6 (50.0%)	-	0.00222	Palermo and Trapani
**2841G > T (E908X)**	c.2722G > T (p.Glu908Ter)	SNV	11 (2.28%)	4 (36.36%)	-	0.00148	Palermo, Agrigento, Caltanissetta and Enna
**5382insC**	c.5266dupC (p.Gln1756Profs)	Duplication	11 (2.28%)	6 (54.54%)	ExAC 0.000156 GnomAD 0.00018	0.00222	Messina
**5149del4 (T1677fs)**	c.5030_5033del (p.Thr1677fs)	Deletion	11 (2.28%)	2 (18.18%)	-	0.00074	Caltanissetta and Palermo
**3347delAG**	c.3226_3227AG [1] (p.Gly1077fs)	Deletion	10 (2.07%)	4 (40.0%)	GnomAD 0.000004	0.00148	Palermo
**916delTT (S220fs)**	c.798_799del (p.Ser267fs)	Deletion	10 (2.07%)	5 (50.0%)	-	0.00185	Palermo
**4023G > T (E1302X)**	c.3904G > T (p.Glu1302Ter)	SNV	8 (1.66%)	6 (75.0%)	-	0.00222	Caltanissetta
**184T > C (L22S)**	c.65T > C (p.Leu22Ser)	SNV	8 (1.66%)	2 (25.0%)	-	0.00074	Palermo
300T > G (C61G)***	c.181T > G (p.Cys61Gly)***	SNV	8 (1.66%)	2 (25.0%)	ExAC 0.000067 GnomAD 0.000032	0.00074	Ragusa and Palermo
**4843delC (P1596fs)**	c.4583del (p.Pro1528fs)	Deletion	7 (1.45%)	2 (28.57%)	-	0.00074	Palermo
**3519G > T (E1134X)**	c.3400G > T (p.Glu1134Ter)	SNV	5 (1.03%)	2 (40.0%)	GnomAD 0.000004	0.00074	Palermo
**3372insA**	c.3253dupA (p.Arg1085Lysfs)	Duplication	5 (1.03%)	1 (20.0%)	-	0.00037	Catania

* Dataset ExAC v1.0; ** Dataset GnomAD v2.1.1. *** This PV is present together with the PV IVS18 + 2T > C (HGVS: c.8331 + 2T > C) (reported in Table S2) in one of two probands showing double heterozygosity for *BRCA1* and *BRCA2*, therefore the total number of *BRCA1* PV cancer patients is 104 and the total number of *BRCA2* cancer patients is 98.

**Table 4:** Type and Frequency of Germline *BRCA2* PVs (class V) and Geographical Area of Origin of *BRCA* Carriers.

BIC NOMENCLATURE	HGVS NOMENCLATURE	TYPE OF *BRCA2* PV	*BRCA2* PV CARRIERS (Patients and Family Members)	*BRCA2* PV Cancer Patients	Allele Frequency (ExAC */GnomAD **)	Allele Frequency (Sicilian Population)	GEOGRAPHICAL AREA
**1466delT (L413fs)**	c.1238del (p.Leu413fs)	Deletion	49 (10.10%)	20 (40.82%)	GnomAD 0.000004	0.00742	Palermo
**6079del4 (S1951fs)**	c.5851_5854del (p.Ser1951fs)	Deletion	17 (3.50%)	8 (47.06%)	-	0.00297	Trapani and Palermo
**6310del5 (E2028fs)**	c.6082_6086del (p.Glu2028fs)	Deletion	16 (3.30%)	5 (31.25%)	ExAC 0.000008 GnomAD 0.000004	0.00185	Trapani
**IVS19 + 1G > A**	c.8487 + 1G > A	SNV	10 (2.07%)	4 (40.0%)	-	0.00148	Messina, Palermo and Caltanissetta
**9326insA**	c.9098_9099insA (p.Gln3034fs)	Duplication	9 (1.85%)	2 (22.22%)	-	0.00074	Palermo and Agrigento
**L2865X**	c.8594T > A (p.Leu2865Ter)	SNV	8 (1.66%)	3 (37.5%)	-	0.00111	Palermo
**V211I**	c.631G > A (p.Val211Ile)	SNV	8 (1.66%)	4 (50.0%)	-	0.00148	Agrigento, Siracusa and Ragusa
**6714del4**	c.6482_6485ACAA [1] (p.Lys2162fs)	Deletion	6 (1.24%)	1 (16.66%)	ExAC 0.000017 GnomAD 0.000009	0.00037	Messina
**9254del5 (Y3009fs)**	c.9026_9030del (p.Tyr3009fs)	Deletion	6 (1.24%)	3 (50.0%)	GnomAD 0.000004	0.00111	Palermo
**6265A > T (K2013X)**	c.6037A > T (p.Lys2013Ter)	SNV	5 (1.03%)	1 (20.0%)	GnomAD 0.000004	0.00037	Erice
**Q2561X**	c.7681C > T (p.Gln2561Ter)	SNV	5 (1.03%)	1 (20.0%)	-	0.00037	Palermo and Messina
**3036del4 (A938fs)**	c.2808_2811del (p.Ala938Profs)	Deletion	5 (1.03%)	2 (40.0%)	ExAC 0.000017 GnomAD 0.000008	0.00074	Caltanissetta
**4512insT**	c.4284dup (p.Gln1429fs)	Duplication	4 (0.82%)	3 (75.0%)	GnomAD 0.000004	0.00111	Palermo
**2070insT**	c.1842dupT (p.Asn615Terfs)	Duplication	4 (0.82%)	1 (25.0%)	-	0.00037	Agrigento
**IVS21 + 4A > G**	c.8754 + 4A > G	SNV	4 (0.82%)	2 (50.0%)	-	0.00074	Catania

* Dataset ExAC v1.0; ** Datase GnomAD v2.1.1.

## Supplementary Materials

The following are available online at https://www.mdpi.com/2072-6694/12/5/1158/s1, Figure S1: Number of genetic testing for BRCA1/2 genes performed on HBOC patients every year at our Centre (1999-2019), Figure S2: Total number of individuals included in the Hereditary Breast/Ovarian Cancer (HBOC) study (1999-2019), Table S1: Other Pathogenic Variants (class V) and Variants of Uncertain Significance (class III) detected in BRCA1 gene, Table S2: Other Pathogenic Variants (class V) and Variants of Uncertain Significance (class III) detected in *BRCA2* gene.
