# Supplementary material for: Hereditary Breast and Ovarian Cancer in Families from Southern Italy (Sicily)—Prevalence and Geographic Distribution of Pathogenic Variants in BRCA1/2 Genes
Source: Cancers (Basel). 2020 May 5;12(5):1158. doi: 10.3390/cancers12051158 (PMC7280980; doi:10.3390/cancers12051158)
Supplement: Supplementary file 1 [file cancers-12-01158-s001.pdf]

Hereditary Breast and Ovarian Cancer in Families from Southern Italy (Sicily)—Prevalence and Geographic Distribution of Pathogenic Variants in *BRCA1*/2 Genes

Lorena Incorvaia, Daniele Fanale, Giuseppe Badalamenti, Marco Bono, Valentina Calò, Daniela Cancelliere, Marta Castiglia, Alessia Fiorino, Alessia Pivetti, Nadia Barraco, Sofia Cutaia and Antonio Russo and Viviana Bazan

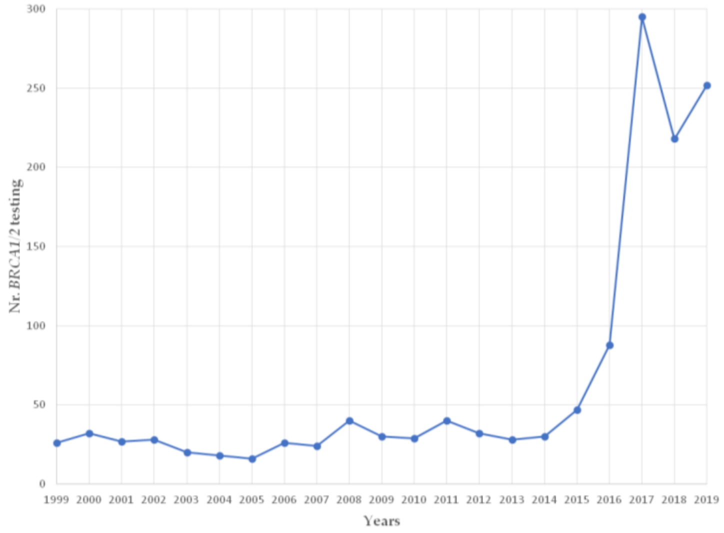

Figure S1. Number of genetic testing for *BRCA1/2* genes performed on HBOC patients every year at our Centre (1999-2019).

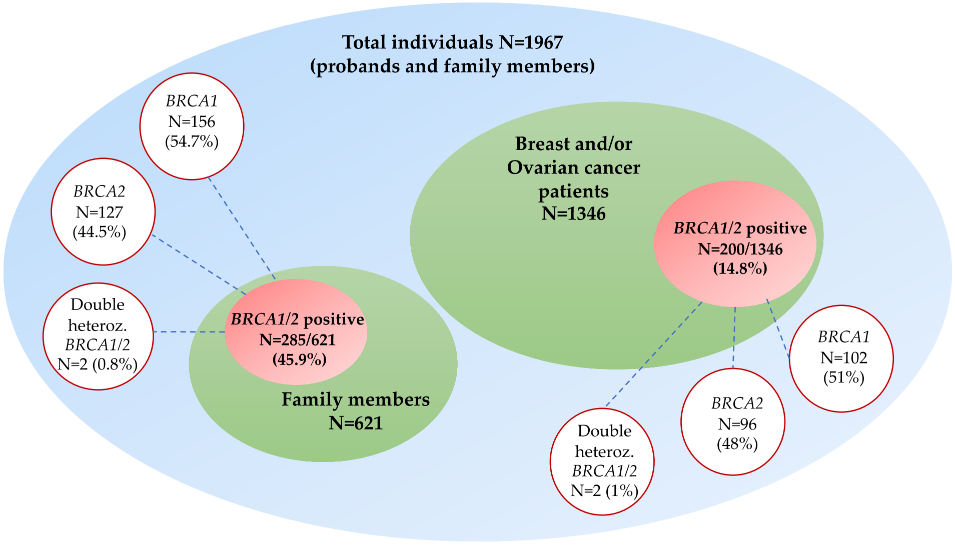

Figure S2. Total number of individuals included in the Hereditary Breast/Ovarian Cancer (HBOC) study (1999-2019). Considering both probands and family members, overall 1967 individuals were genetically tested for *BRCA1/2* PVs. Our analysis showed that 485 (24.6%) out of 1967 subjects (probands and family members) harbour a germline *BRCA1/2* PV. 200 (14.8%) out of 1346 were HBOC patients and 285 (45.9%) out of 621 were family members harbouring a *BRCA1/2* PV.

Table S1. Other Pathogenic Variants (class V) and Variants of Uncertain Significance (class III) detected in *BRCA1* gene.

GENE	BIC NOMENCLATURE	HGVS NOMENCLATURE	VARIANT CLASSIFICATION	CANCER PATIENTS	CARRIERS (patients and family members)
BRCA1	3663C>T (Q1182X)*	c.3544C>T (p.Gln1182Ter)*	V	3	5
	5242C>A (A1708E)	c.5123C>A (p.Ala1708Glu)	V	1	4
	1103insC	c.984_985insC (p.Asn329fs)	V	1	4
	185insA	c.66dupA (p.Glu23Argfs)	V	1	4
	IVS8+2T>A	c.547+2T>A	V	1	4
	185delAG	c.66_67AG[1] (p.Glu23fs)	V	1	3
	1001delA (D295fs)	c.882del (p.Asp295fs)	V	1	3
	236delTG	c.115_116TG[1] (p.Cys39_Asp40delinsTer)	V	1	2
	3385delT (L1042fs)	c.3266del (p.Leu1089fs)	V	1	2
	290delG (P58fs)	c.171del (p.Pro58fs)	V	1	2
	2388delG (V757fs)	c.2269del (p.Val757fs)	V	1	2
	1135delA (K292fs)	c.1016del (p.Lys339fs)	V	1	2
	5190insA	c.5071dupA (p.Thr1691Asnfs)	V	1	1
	5215G>A (R1699Q)	c.5096G>A (p.Arg1699Gln)	V	1	1
	3486G>T (D1123Y)	c.3367G>T (p.Asp1123Tyr)	III	1	1
	2000C/G	c.1881C>G (p.Val627=)	III	2	2
	K1487R	c.4460A>G (p.Lys1487Arg)	III	1	1
	IVS11+3A>G	c.4096+3A>G	III	1	1
	E1352K	c.4054G>A (p.Glu1352Lys)	III	2	2
	S1580F	c.4739C>T (p.Ser1580Phe)	III	1	1
	IVS7+36C>T	c.441+36C>T	III	1	1
	M297L	c.889A>C (p.Met297Leu)	III	1	1
	N1355del	c.4063_4065delAAT (p.Asn1355del)	III	1	1
	E23Q	c.67G>C (p.Glu23Gln)	III	1	1
	R1634G	c.4900A>G (p.Arg1634Gly)	III	1	1
	T248P	c.742A>C (p.Thr248Pro)	III	1	1

* This PV is present together with the PV IVS13-2A>T (HGVS: c.7008-2A>T) (reported in Table S2) in one of two probands showing double heterozygosity for *BRCA1* and *BRCA2*, therefore the total number of *BRCA1* PV cancer patients is 104 and the total number of *BRCA2* cancer patients is 98.

Table S2. Other Pathogenic Variants (class V) and Variants of Uncertain Significance (class III) detected in *BRCA2* gene.

GENE	BIC NOMENCLATURE	HGVS NOMENCLATURE	VARIANT CLASSIFICATION	CANCER PATIENTS	CARRIERS (patients and family members)
BRCA2	2041delA (I605fs)	c.1813del (p.Ile605fs)	V	3	4
	IVS13-2A>T*	c.7008-2A>T*	V	3	4
	IVS18+2T>C**	c.8331+2T>C**	V	2	4
	3386T>G (L1053X)	c.3158T>G (p.Leu1053Ter)	V	2	4
	7218del5 (I2330fs)	c.6990_6994del (p.Ile2330fs)	V	2	4
	6252insG	c.6024dupG (p.Gln2009Alafs)	V	2	4
	6187C>T (Q1987X)	c.5959C>T (p.Gln1987Ter)	V	2	4
	5946delCTCT	c.5718_5719CT[2] (p.Leu1908fs)	V	2	4
	7708C>T (R2494X)	c.7480C>T (p.Arg2494Ter)	V	2	4
	983del4	c.751_754ACAG[1] (p.Asp252fs)	V	2	4
	IVS5-2A>G	c.476-2A>G	V	2	3
	2881del4 (D885fs)	c.2653_2656del (p.Asp885fs)	V	2	3
	9481insA	c.9253dupA (p.Thr3085Asnfs)	V	2	3
	5823delAT	c.5593_5594AT[1] (p.Phe1866fs)	V	2	3
	9376C>T (Q3050X)	c.9148C>T (p.Gln3050Ter)	V	2	3
	9683delAG	c.9453_9454AG[1] (p.Glu3152fs)	V	2	3
	6503delTT (L2092fs)	c.6275_6276del (p.Leu2092fs)	V	1	3
	5991delT (F1921fs)	c.5763del (p.Phe1921fs)	V	1	3
	6352C>T (Q2042X)	c.6124C>T (p.Gln2042Ter)	V	1	2
	5302insA	c.5073dupA (p.Trp1692Metfs)	V	1	2
	V1756E	c.5267T>A (p.Val1756Glu)	III	1	1
	K100E	c.298A>G (p.Lys100Glu)	III	1	1
	S2697R	c.8091C>A (p.Ser2697Arg)	III	1	1
	R3384G	c.10150C>G (p.Arg3384Gly)	III	2	2
	S2807P	c.8419T>C (p.Ser2807Pro)	III	1	1
	K1517R	c.4550A>G (p.Lys1517Arg)	III	1	1

T1426I	c.4277C>A (p.Thr1426Ile)	III	1	1
I1173F	c.3517A>T (p.Ile1173Phe)	III	1	1
8490T>G (H2754Q)	c.8262T>G (p.His2754Gln)	III	1	1
I1831T	c.5492T>C (p.Ile1831Thr)	III	2	2
P2798R	c.8393C>G (p.Pro2798Arg)	III	1	1
10067C>A (P3280H)	c.9839C>A (p.Pro3280His)	III	1	1
M1890T	c.5669T>C (p.Met1890Thr)	III	1	1
T567I	c.1700C>T (p.Thr567Ile)	III	1	1
692G>C (R115T)	c.464G>C (p.Arg155Thr)	III	1	1
L3101P	c.9302T>C (p.Leu3101Pro)	III	1	1
P2767S	c.8299C>T (p.Pro2767Ser)	III	1	1
E1254D	c.3762G>T (p.Glu1254Asp)	III	1	1
1997T>G (F590C)	c.1769T>G (p.Phe590Cys)	III	1	1
5656G>A (V1810I)	c.5428G>A (p.Val1810Ile)	III	1	1
3209C>T (A994V)	c.2981C>T (p.Ala994Val)	III	1	1
K1888R	c.5663A>G (p.Lys1888Arg)	III	1	1
F1506L	c.4516T>C (p.Phe1506Leu)	III	1	1

* This PV is present together with the PV 3663C>T (HGVS: c.3544C>T; p.Gln1182Ter) (reported in Table S1) in one of two probands showing double heterozygosity for *BRCA1* and *BRCA2*. ** This PV is present together with the PV 300T>G (HGVS: c.181T>G; p.Cys61Gly) (reported in Table 3) in one of two probands showing double heterozygosity for *BRCA1* and *BRCA2*. Therefore, the total number of *BRCA1* PV cancer patients is 104 and the total number of *BRCA2* cancer patients is 98.
